# The Citrus Laccase Gene *CsLAC18* Contributes to Cold Tolerance

**DOI:** 10.3390/ijms232314509

**Published:** 2022-11-22

**Authors:** Xiaoyong Xu, Yueliang Zhang, Mengge Liang, Weiwen Kong, Jihong Liu

**Affiliations:** 1 School of Horticulture and Plant Protection, Yangzhou University, Yangzhou 225009, China; 2 Key Laboratory of Horticultural Plant Biology (MOE), College of Horticulture and Forestry Science, Huazhong Agricultural University, Wuhan 430070, China

**Keywords:** citrus, laccase, cold stress, lignin

## Abstract

Plant laccases, as multicopper oxidases, play an important role in monolignol polymerization, and participate in the resistance response of plants to multiple biotic/abiotic stresses. However, little is currently known about the role of laccases in the cold stress response of plants. In this study, the laccase activity and lignin content of *C. sinensis* leaves increased after the low-temperature treatment, and cold treatment induced the differential regulation of 21 *CsLACs*, with 15 genes being upregulated and 6 genes being downregulated. Exceptionally, the relative expression level of *CsLAC18* increased 130.17-fold after a 48-h treatment. The full-length coding sequence of *CsLAC18* consists of 1743 nucleotides and encodes a protein of 580 amino acids, and is predominantly expressed in leaves and fruits. CsLAC18 was phylogenetically related to AtLAC17, and was localized in the cell membrane. Overexpression of *CsLAC18* conferred enhanced cold tolerance on transgenic tobacco; however, virus-induced gene silencing (VIGS)-mediated suppression of *CsLAC18* in *Poncirus trifoliata* significantly impaired resistance to cold stress. As a whole, our findings revealed that *CsLAC18* positively regulates a plant’s response to cold stress, providing a potential target for molecular breeding or gene editing.

## 1. Introduction

Citrus is one of the most important fruit trees with high economic and nutritional value. The statistics of the Food and Agriculture Organization (FAO) reveal that the worldwide citrus cultivated area exceeds 10.07 million hectares with a yield of more than 158.49 million tons (FAOSTAT, 2020) [1]. Being sessile organisms, citrus often encounters diverse environmental stresses, including biotic and abiotic stresses. Most notably, cold stress is among the most devastating abiotic factor limiting the growth, development, and geographical distribution of citrus [2]. Therefore, the breeding of cold-tolerant citrus cultivars is a practical approach to mitigate the effects of chilling stress conditions. Unfortunately, traditional citrus breeding has often been limited due to high degrees of heterozygosity, polyembryony (nucellar embryos), and male and/or female sterility [3]. Transgenic breeding provides an alternative approach to address this issue. Therefore, mining and identifying potential genes involved in cold stress tolerance is necessary for citrus.

Laccases (p-diphenol–dioxygen oxidoreductase, EC 1.10.3.2, LACs) belong to multicopper oxidases that are involved in the oxidation of a wide range of phenolic compounds concomitant with the reduction of molecular oxygen (O_2_) to water (H_2_O) [4]. LACs are broadly found in insects, bacteria, fungi, and plants since Yoshida first described them in 1883 [5]. Previous studies have suggested that plant LACs play a pivotal role in monolignol polymerization, anthocyanin biosynthesis, seed development, and biotic/abiotic stress responses [6,7,8]. Among them, the role of LACs in environmental stress responses has attracted the most attention. Generally, overexpression of *LACs* can increase the resistance of plants to different pathogens and insects. For instance, overexpression of cotton *GhLAC1*, *GhLAC15*, and *EuLAC1* from *Eucommia ulmoides* enhanced lignin accumulation, thereby improving resistance to *Botrytis cinerea*, *Verticillium dahlia*, and *Botrytis cinerea*, respectively [9,10,11]. Surprisingly, *GhLAC1* RNAi transgenic plants displayed increased tolerance to *Verticillium dahliae* and cotton bollworm, owing to the accumulation of jasmonic acid and secondary metabolites [12].

Interestingly, *LACs* are notably expressed under multiple abiotic stresses, including salt, drought, low temperatures, and heavy metal toxicity [13]. Furthermore, overexpression of rice *OsChI1* (Os01g61160, *LAC*) and *OsLAC10* increased salt/drought tolerance and copper tolerance in *Arabidopsis*, respectively [14,15]. Notably, laccase expression is usually upregulated by low-temperature stress. For example, *OsChI1* from rice (*Oryza sativa*), *DcLAC1* from carrot (*Daucus carota*), four *LACs* from eggplant (*Solanum melongena*), and five *LACs* from orange (*Citrus sinensis*) exhibited upregulated expression after low-temperature treatment [15,16,17,18]. However cold treatment (4 °C) led to the downregulation of *SmLAC12* [17]. Additionally twenty-five *PbLACs* of pear (*Pyrus bretschneideri*) showed distinct expression patterns following cold treatment [19]. The above-mentioned studies suggest that *LACs* are involved in the response to low-temperature stress, and the role of *LACs* needs to be further elucidated by functional studies.

A previous study in our lab showed that there are 24 LAC family members in the *Citrus sinensis* genome [18]. Thus, the present study aimed to mine and identify the potential key *CsLACs* involved in cold stress tolerance. Firstly, we measured the laccase activity and lignin content of *C. sinensis* leaves after cold treatment, and investigated the expression patterns of 24 *CsLACs* via quantitative real-time PCR (qRT-PCR) analysis. Secondly, we performed the cloning, sequence analysis, and subcellular localization of *CsLAC18*. Finally, we explored the function of *CsLAC18* associated with cold stress through both VIGS and overexpression in *P. trifoliata* and tobacco, respectively. Altogether, we, for the first time, provide evidence that LACs play an important role in the response to cold stress.

## 2. Results

### 2.1. Changes in Laccase Activity and Lignin Content in Response to Cold Stress in C. sinensis

**Figure 1:**
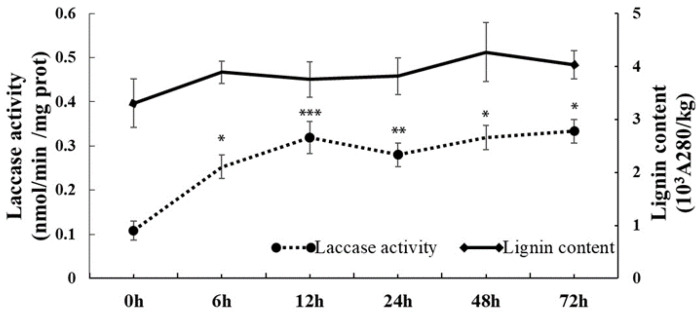
Changes in the laccase activity and lignin content of *C. sinensis* leaves after cold treatment. Asterisks indicate significant difference between control (0 h) and cold treatment (* *p* < 0.05, ** *p* < 0.01, *** *p* < 0.001).

To investigate whether the laccases are involved in the cold stress response, we measured laccase activity under low-temperature stress. After the *C. sinensis* seedlings were cold-treated at 4 °C for 12 h, the laccase activity of *C. sinensis* leaves showed a progressive increase, followed by a minor decline at 24 h, and continued to rise until the end of the experiment (Figure 1). Since laccases participate in lignin formation, we simultaneously estimated the lignin content during the low-temperature treatment. The dynamic changes in lignin content were similar to those of the laccase activity under cold stress, except for a minor decrease at 12 or 72 h. Furthermore, correlation analyses indicated a significant correlation between laccase activity and lignin content (Supplementary Figure S1). These results indicate that laccases might be involved in the cold stress response via the regulation of lignin biosynthesis in *C. sinensis*.

### 2.2. Expression Analysis of CsLACs in Response to Cold Treatment

**Figure 2:**
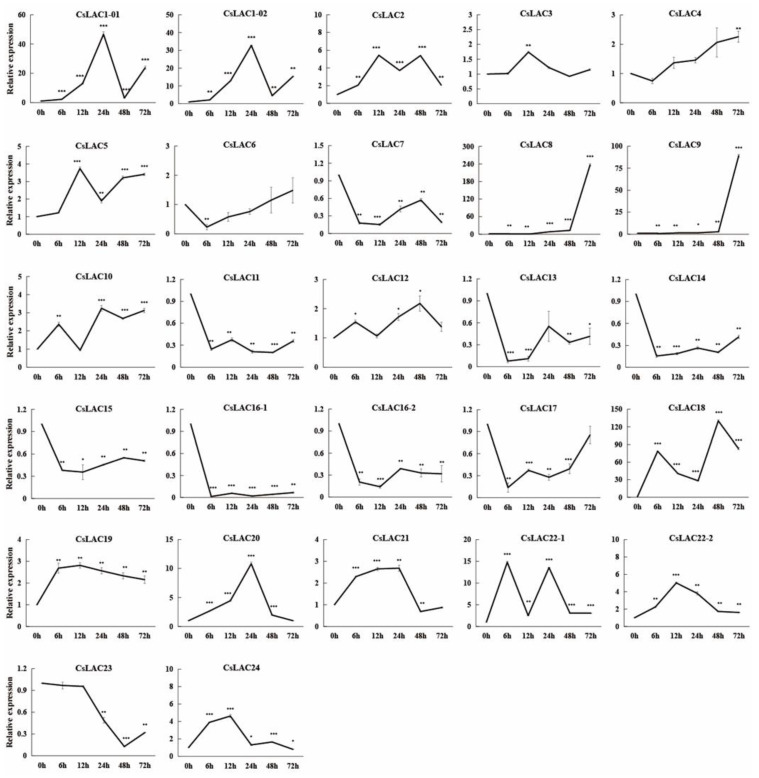
Expression patterns of *CsLACs* under cold stress treatment. Asterisks indicate significant difference between control (0 h) and cold treatment (* *p* < 0.05, ** *p* < 0.01, *** *p* < 0.001).

It is well known that laccases are encoded by multiple genes. Our previous studies identified 24 laccase genes from the *C. sinensis* genome [18]. To mine the key laccase genes in response to cold stress in *C. sinensis*, the relative expression levels of 24 *CsLACs* were assessed after cold treatment by qRT-PCR analysis. As shown in Figure 2, cold treatment induced the differential regulation of 21 *CsLACs*, with 15 genes being upregulated and 6 genes being downregulated (Figure 2). Of these, a more than 100-fold upregulation was found for two *CsLACs* (*CsLAC8* and *CsLAC18*). In particular, the relative expression of *CsLAC18* increased sharply just after 6 h (78.69 times of the initial value) and reached the maximal value (130.17 times) after 48 h. Therefore, we speculated that CsLAC18 is a key laccase gene in the response to cold stress in *C. sinensis.*

### 2.3. Cloning, Sequence Analysis, and Subcellular Localization of CsLAC18

Based on the sequence information from the citrus genome database, we cloned the full-length coding sequences of *CsLAC18* from *C. sinensis*, which consists of 1743 nucleotides (Supplementary Figure S2), and encoded a protein of 580 amino acids with a predicted molecular mass of 64.02 kDa and an isoelectric point of 9.10. Simple Modular Architecture Research Tool (SMART) analysis showed that CsLAC18 had three characteristic domains (Cu_oxidase, Cu_oxidase_2, and Cu_oxidase_3). In addition, a predicted signal peptide composed of 30 amino acids was observed at the N terminal (Supplementary Figure S3). According to public transcriptomic data, *CsLAC18* is predominantly expressed in leaves and fruits (Supplementary Table S1).

**Figure 3:**
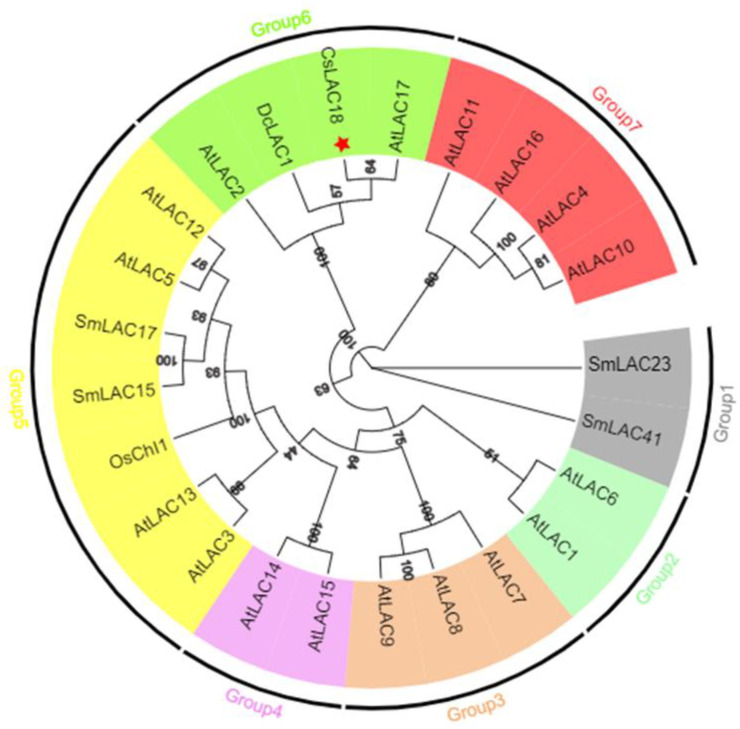
Phylogenetic tree analysis of *LACs* in *C. sinensis* and other plants. The phylogenetic tree was constructed using MEGA X by the NJ method with p-distance substitution model (gamma = 1) and 1000 bootstrap replicates. The red star indicates the location of CsLAC18.

To reveal the phylogenetic relationship between CsLAC18 and homologous proteins from *Arabidopsis thaliana* and other plants, we constructed an unrooted maximum likelihood phylogenetic tree using MEGA X software (Figure 3). According to the phylogenetic tree, LACs were clustered into seven groups, and CsLAC18 was classified into Group 6 with AtLAC17 (81% identity), DcLAC1 (79% identity) and AtLAC2 (72% identity).

**Figure 4:**
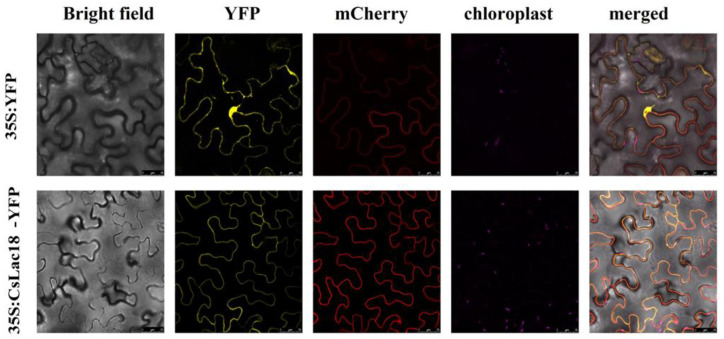
Subcellular localization of CsLAC18. Images of the representative tobacco epidermal cells from leaves transformed with 35S:YFP or 35S:CsLAC18-YFP fusion protein were taken under bright field, yellow fluorescence (YFP), red fluorescence (mCherry), chloroplast autofluorescence, and merged. Scale bars = 25 μm.

The subcellular localization prediction by Plant-mPLoc server showed that CsLAC18 was localized in the cell membrane. In order to verify this assumption, the CsLAC18 full-length cDNA without stop codon was fused to the Yellow Fluorescent Protein (YFP) gene in-frame under the control of the cauliflower mosaic virus (CaMV) 35S promoter. Confocal microscopy analysis showed that the YFP signal was distributed throughout the cell when the vector of 35S:YFP was transiently expressed in tobacco leaves (Figure 4). However, the fusion protein 35S:CsLAC18-YFP was exclusively expressed in the cell membrane, indicating that CsLAC18 was localized in the cell membrane.

### 2.4. Overexpression of CsLAC18 Confers Enhanced Cold Tolerance

**Figure 5:**
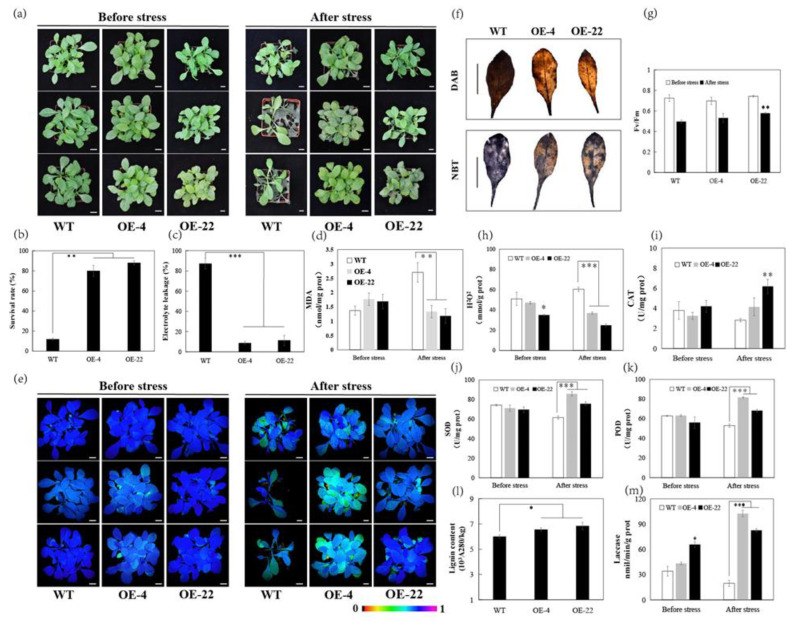
Overexpression of *CsLAC18* enhanced cold tolerance in transgenic tobacco plants. (**a**) The phenotype of wild-type (WT) and transgenic lines (4 and 22) before and after cold treatment; (**b**,**c**) survival rate (**b**) and electrolyte leakage (EL) level (**c**) in the WT and transgenic lines, measured after the cold treatment; (**d**,**e**) malondialdehyde (MDA) (**d**) content and chlorophyll fluorescence imaging (**e**) before and after cold treatment; (**f**) DAB and NBT staining of the leaves under cold stress; (**g**–**k**,**m**) Fv/Fm ratio (**g**), H_2_O_2_ content (**h**), CAT activity (**i**), SOD activity (**j**), POD activity (**k**), and laccase activity (**m**) of WT and transgenic lines before and after the freezing treatment; (**l**) lignin content of WT and transgenic lines. Asterisks indicate significant difference between the WT and the transgenic tobacco plants (* *p* < 0.05, ** *p* < 0.01, *** *p* < 0.001). Scale bars = 1 cm (**a**,**e**,**f**).

To understand the role of *CsLAC18* in the cold stress response, we overexpressed *CsLAC18* in tobacco by *Agrobacterium*-mediated transformation, and all the transgenic lines were confirmed by PCR analysis (Supplementary Figure S4). Among the transgenic lines, two independent overexpression lines (OE-4 and OE-22) were chosen for further characterization. The 4-week-old wild-type (WT) and overexpression plants exhibited no apparent morphological difference without stress treatments (Figure 5a). After 12 h incubation at −4 °C, compared with the WT, the overexpression lines suffered from less wilting (Figure 5a). Following recovery for 7 days, the overexpression lines (4 and 22) had higher survival rates (89% and 84%) than the WT (15%) (Figure 5b). Electrolyte leakage (EL) and malondialdehyde (MDA) are widely used as indicators of damage caused by stress. The EL and MDA levels were significantly lower in the overexpression lines than in the WT under cold stress (Figure 5c,d), suggesting that the overexpression lines suffered less membrane damage than the WT. Moreover, overexpression lines showed brighter chlorophyll fluorescence and higher Fv/Fm ratios than the WT after low-temperature treatment (Figure 5e,g). These data indicate that overexpression of *CsLAC18* improves the chilling stress tolerance of transgenic tobacco plants.

Furthermore, the degree of oxidative stress and the antioxidant systems between WT and the overexpression lines was evaluated. According to Figure 5f,h, ROS accumulation was lower in the overexpression lines than in the WT after the chilling treatment. By contrast, the activities of the antioxidant enzymes catalase (CAT), superoxide dismutase (SOD), and peroxidase (POD) were significantly higher in the overexpression lines than in the WT after a 12 h cold exposure (Figure 5i–k). In addition, the activities of laccases were significantly higher in the overexpression lines than in the WT before and after cold treatment (Figure 5m). Meanwhile, the overexpression lines showed higher lignin content compared with the WT (Figure 5l). These results demonstrate that overexpression of *CsLAC18* significantly improved the endogenous antioxidant activity.

### 2.5. Silencing of CsLAC18 in Poncirus Trifoliata increases Cold Sensitivity

**Figure 6:**
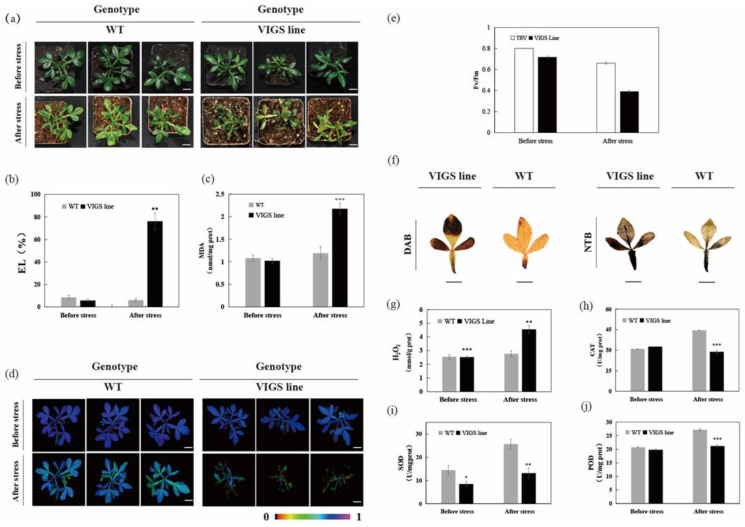
Silencing of *CsLAC18* by VIGS causes enhanced cold sensitivity in *Poncirus trifoliata*. (**a**–**e**,**g**–**j**) Phenotype (**a**), EL (**b**), MDA content (**c**), chlorophyll fluorescence imaging (**d**), Fv/Fm ratio (**e**), H_2_O_2_ level (**g**), CAT activity (**h**), SOD activity (**i**), and POD activity (**j**) of control plants (TRV) and VIGS plants (TRV-CsLAC18) before and after the cold treatment (12 h at −4 °C). (**f**) DAB and NBT staining of the leaves of trifoliate orange under cold stress. Asterisks indicate significant difference between the WT and the VIGS lines (* *p* < 0.05, ** *p* < 0.01, *** *p* < 0.001). Scale bars = 1 cm (**a**,**d**,**f**).

To further confirm the role of *CsLAC18* in cold tolerance, we silenced *CsLAC18* in trifoliate orange by VIGS. In ten VIGS plants, the *CsLAC18* transcript level was downregulated by 28% to 67%, compared with the TRV control plants (Supplementary Figure S5). Under normal growth conditions, the VIGS lines (3, 24 and 35) showed the same phenotype as the TRV control plants (Figure 6a). However, freezing treatment (−4 °C for 12 h) induced more severe wilting in the VIGS lines compared with the TRV control plants. Meantime, in comparison with the TRV control plants, high EL and MDA levels, weak chlorophyll fluorescence, and a low Fv/Fm ratio were observed in the VIGS lines (Figure 6b–e). Furthermore, both histochemical staining and quantitative measurement demonstrated that ROS accumulation was higher in the VIGS lines than in the WT after chilling treatment (Figure 6f,g). At the same time, the VIGS line showed lower CAT, SOD, and POD activities than the control under cold stress (Figure 6h,j). Taken together, these results indicate that silencing of *CsLAC18* in trifoliate orange results in increased susceptibility to cold stress.

## 3. Discussion

The laccase-mediated synthesis of lignin plays an important role in regulating plant growth and development as well as resistance to biotic/abiotic stresses [8,20]. In 1995, Anderson et al. reported the earliest observation of elevated lignin content during acclimation to chilling in the mesocotyls of maize seedlings [21]. Subsequently, the elevated lignin content induced by low temperature was reported in winter wheat, sugarcane, poplar, chickpea, *Camellia oleifera*, and tobacco [22,23,24,25,26,27], suggesting that the accumulation of lignin may be an important strategy for adapting plants to cold stress [28]. Here, we determined the laccase activity and lignin content of *C. sinensis* during cold treatment (Figure 1). Our results showed that lignin accumulation occurred in leaves, while the lignin content also showed positive and significant correlation with laccase activity. Considering laccases encoded by multiple genes, the potential key *CsLACs* involved in cold stress tolerance remains to be determined.

In this study, 15 *CsLACs* showed upregulated expression patterns following low-temperature treatment. Among them, *CsLAC18* was the most significantly upregulated gene (Figure 2). This low-temperature-responsive expression was also observed in other plants, such as rice, carrot, pear, and eggplant [15,16,17,19]. Additionally, our previous study revealed that the LTR motif (*cis*-acting element involved in low-temperature responsiveness) was predicted to be located in the promoter regions of eight chilling-responsive *CsLACs* [18]. Moreover, low-temperature *cis*-elements were found in the promoters of 8 *LACs* from *Brassica napus* [29], 11 *LACs* from eggplant [17], 21 *LACs* from switchgrass [30], 12 *LACs* from tea plant [31], 45 *LACs* from wheat [32], and 16 *LACs* from peach [33]. Therefore, the above results indicate that low-temperature *cis*-elements are essential for the *LACs* to respond to cold stress. In the future, promoter deletion analysis will be used to determine the function of the predicted low-temperature *cis*-elements of *CsLACs*.

The full length of *CsLAC18* was cloned from *C. sinensis*, and CsLAC18 was presumed to contain three characteristic domains (Supplementary Figures S2 and S3). CsLAC18 was localized in the cell membrane, consistent with earlier reports [15]. Notably, CsLAC18 was phylogenetically closely related to AtLAC17 (Figure 3). Using reverse genetics, Berthet et al. (2011) demonstrated that *AtLAC17* is involved in lignin polymerization by affecting the deposition of G lignin units in the interfascicular fibers [34]. Interestingly, three laccase genes (*BdLAC5*, *EuLAC1*, and *SofLAC*), which are closely related to lignin-specific *AtLAC17*, were confirmed to participate in lignification [9,35,36]. Therefore, *CsLAC18* may have a similar function to *AtLAC17*. In addition, cold-stress-responsive *LACs* were clustered in different branches. For instance, *SmLAC23* and *SmLAC41* were categorized into Group 1; *OsChI1*, *SmLAC15*, and *SmLAC17* were assigned into Group 5; and *DcLAC1* and *CsLAC18* were classified into Group 6 (Figure 3). It can be speculated that these cold-stress-responsive genes might work in different pathways or with other mechanisms.

Our results showed that the overexpression of *CsLAC18* in the tobacco increased the resistance to cold stress, while silencing of *CsLAC18* by VIGS in trifoliate oranges greatly decreased tolerance to cold stress (Figure 5 and Figure 6). Moreover, upregulation of *CsLAC18* in the tobacco led to increased laccase activity and lignin content. We speculate that there are two possible reasons for these findings. Firstly, it has been widely demonstrated that lignin plays a significant function in plants’ adaptation to their environment, and induced ‘stress lignin’ share certain physical characteristics that allow plants to adapt to stress conditions [20]. In the present study, it is possible that the CsLAC18–laccase–lignin module prevents plants from cellular damages caused by cold stress via the modulation of cell wall rigidity [28]. Secondly, the previous studies found that laccases were involved in the regulation of secondary metabolite biosynthesis in plants and some of the secondary metabolites were implicated in the responses to various environmental stresses [6,12,37]. For example, Hu et al. reported that the inhibition of *GhLac1* expression resulted in a redirection of metabolic flux in the phenylpropanoid pathway and the accumulation of secondary metabolites that conferred resistance of cotton plants to various biotic stresses [12]. Therefore, we hypothesized that overexpression of *CsLAC18* might induce the production of certain secondary metabolites that activate the enzymatic antioxidant system after cold stress.

## 4. Materials and Methods

### 4.1. Plant Materials and Cold Treatment

In this study, Valencia sweet orange (*C. sinensis*) and trifoliate orange (*Poncirus trifoliata*) were used as the main research materials. Sweet orange is one of the most economically important tree fruit crops in the world. Trifoliate orange, a widely used rootstock for citrus production, is extremely cold-hardy, and it is used as a valuable material for the identification of cold-resistant candidate genes.

Seeds of Valencia sweet orange and trifoliate orange were surface sterilized for 20 min with sodium hypochlorite solution (10%) and then washed three times using distilled water, followed by inoculation on MT medium containing 30 g L^−1^ sucrose. For cold stress treatment, 8-week-old seedlings were maintained at 4 °C for 0, 6, 12, 24, 48, and 72 h under growth chamber with a photoperiod of 16 h light (60–70 µmm^−2^ s^−1^)/8 h dark, after which the leaves were harvested and immediately frozen in liquid nitrogen. One part was used for RNA extraction and the other part for physiological indexes’ measurement. Three biological replicates were performed, and each replicate contained the leaves from five seedlings.

### 4.2. Determination of Laccase Activity and Lignin Content

The laccase activity assay was performed using commercial kits (Suzhou Comin Biotechnology Co. Ltd., Suzhou, China) according to instructions provided by the manufacturer. The laccase activity was determined by the oxidation of ABTS, which was quantified at 420 nm. The level of laccase activity was expressed as nmol min^−1^ mg^−1^ protein. Lignin content analysis was carried out based on previous reports [38]. Frozen tissue powder was homogenized in 10 mL of washing buffer (0.5% Triton X-100, 100 mM K_2_HPO_4_/KH_2_PO_4_, 0.5% PVP, pH 7.8). The sample was stirred at room temperature for 40 min and then centrifuged for 25 min at 8000× *g*. The resulting pellet was resuspended and washed twice in washing buffer as indicated above. Then the pellet was washed four times in 100% methanol. The washed precipitate was dried in an oven at 80 °C overnight. Ten milligrams of the dried extract residue were suspended in 1.0 mL of 2.0 M HCl and 0.1 mL of thioglycolic acid. The suspension was kept in boiling water bath for 8 h, cooled on ice for 5 min, and then centrifuged at 8000× *g* for 30 min at 4 °C. The pellet was washed with distilled water and resuspended in 2.0 mL 1.0 M NaOH. The solution was gently agitated for 16 h at room temperature and then centrifuged for 25 min at 12,000× *g*. The supernatant (0.5 mL) was taken and mixed with 0.1 mL of concentrated HCl. The precipitated lignin thioglycolic acid was then dissolved in 1 mL of 1 M NaOH. The absorption value at 280 nm was detected using a UV spectrophotometer.

### 4.3. RNA Extraction and Quantitative Real-Time PCR Analysis

Total RNA was extracted using Trizol reagent (TaKaRa, Dalian, China), following the manufacturer’s instructions. The quality of the RNA was examined by gel electrophoresis and Bioanalyzer (Agilent2100). The first strand cDNA was performed by TransScript Reverse transcriptase (TaKaRa, Dalian, China), following the manufacturer’s instructions. Data were analyzed using the β-actin expression as a reference. The gene-specific primers used for the qRT-PCR are shown in Supplementary Table S2. qRT-PCR was carried out with CFX96 real-time PCR machine (BIO-RAD, Hercules, CA, USA). The reaction system and procedure of qPCR was according to our previous report [18]. The 2^−ΔΔCT^ Ct method was used to calculate the relative gene expression levels between the samples.

### 4.4. Cloning and Sequence Analysis of CsLAC18

According to the sequence of *CsLAC18* from the genome of *C. sinensis* (http://citrus.hzau.edu.cn/ accessed on 1 May 2019), a pair of primers (Forward: 5′-ATGGGAGCTTCTCTTCTTCGATC-3′, Reverse: 5′-TCAGCACTGAGGAAGATCTG-3′) were designed and used to amplify the full-length coding sequence (CDS) of *CsLAC18* from *C. sinensis*. The *CsLAC18* conserved domains were identified in SMART database (http://smart.embl-heidelberg.de/ accessed on 3 August 2022). The *CsLAC18* expression data for different tissues were retrieved from Citrus Pan-genome to Breeding Database (http://citrus.hzau.edu.cn/ accessed on 20 August 2022). Multiple sequence alignments were made using ClustalW with the amino acid sequences of laccase proteins. The phylogenetic tree was constructed using MEGA X by neighbor-joining algorithm with bootstrap replication of 1000 times.

### 4.5. Subcellular Localization Analysis of CsLAC18

Subcellular localization analysis was performed as in previous report [39]. The coding region of *CsLAC18* without a termination codon was amplified and cloned into the 101LYFP containing the YFP reporter gene, yielding a fusion protein vector 35S:CsLAC18-YFP. The construct and empty vectors were transiently transformed into tobacco (*Nicotiana benthamiana*) leaves. Subcellular localization of the target protein was observed by a confocal laser scanning microscope (Leica TCS-SP8, Wetzlar, Germany).

### 4.6. Vector Construction and Plant Transformation

The full-length CDS of *CsLAC18* was ligated into pBI121-35S to generate the pBI121-35S: CsLAC18 overexpression construct and then transferred into *Agrobacterium tumefaciens* strain GV3101 by heat shock. *Agrobacterium*-mediated leaf disk transformation of tobacco (*Nicotiana tabacum*) was performed similarly to previous report [40]. The positively transformed tobacco explants were selected on MS medium with 100 mg/L kanamycin and 400 mg/L carbenicillin. The regenerated plants were verified by genomic PCR with specific primers, while expression levels of *CsLAC18* in the transgenic lines were evaluated based on qRT-PCR. T2 lines were used for subsequent experiments.

### 4.7. Virus-Induced Gene Silencing

VIGS-mediated gene silencing of *CsLAC18* was performed as our previous report [41]. A 450 bp fragment of *CsLAC18* was cloned into the tobacco rattle virus-based vector 2 (TRV2) to generate the pTRV2-CsLAC18 vector using the *Bam*HI and *Sma*I sites. The vectors pTRV1, pTRV2, and pTRV2-CsLAC18 were transformed into *Agrobacterium tumefaciens* strain GV3101 using the heat shock method. The germinating seeds of *P. trifoliata* with about 2 cm emerging shoots were infiltrated with the bacterial solutions and then the seedlings were washed with ddH_2_O and dehydrated with filter paper. The seedlings were placed in a controlled growth chamber (Percival, IA, USA) for four weeks. Fully expanded leaves of the seedlings were harvested for genomic PCR and qRT-PCR analyses, and the seedlings that exhibited low expression level of *CsLAC18* were selected for further analysis.

### 4.8. Cold Tolerance Assays, Physiological Measurements and Histochemical Staining

For cold tolerance assay, 4-week-old transgenic tobacco plants or VIGS *P. trifoliata* plants, together with their corresponding WT counterparts were treated for 12 h at −4 °C. Growth performance and chlorophyll fluorescence imaging of the plants were measured before and after the treatment, while leaves were sampled for histochemical staining and physiological assay. The maximum quantum efficiency of photosystem II (Fv/Fm) was determined by Imaging WinGegE software and electrolyte leakage (EL) was evaluated by the method described previously [42]. The H_2_O_2_ levels, MDA contents, SOD, POD, and CAT activities were assessed with corresponding assay kits (Nanjing Jiancheng Bioengineering Institute, Nanjing, China). H_2_O_2_ assay was based upon the oxidative polymerization of molybdic acid into a complex compound, which can be quantified at 405 nm. MDA assay was based upon the thiobarbituric acid (TBA) method and calculated by reading the absorbance of TBA-reactive substances in the supernatant at 532 nm. The H_2_O_2_ or MDA levels were presented as mmol g^−1^ protein or nmol mg^−1^ protein, respectively. SOD activity was assayed by monitoring the inhibition of the photochemical reduction of nitro blue tetrazolium. One unit of SOD was qualified as the enzyme activity that inhibits the reduction of NBT to blue formazan by 50%. POD activity was determined based on its ability to catalyze a reaction of hydrogen peroxide. One unit of POD activity was qualified as the amount of enzyme needed to catalyze 1 µg of substrate present in the homogenate per minute. CAT activity was assayed by measuring H_2_O_2_ decomposition at 405 nm. One unit of CAT activity was qualified as 1 mg of tissue proteins that consumed 1 µmol H_2_O_2_ for 1 s. In situ accumulation of H_2_O_2_ and O^2−^ were examined by histochemical staining with diaminobenzidine (DAB) and nitro blue tetrazolium (NBT), respectively [43].

### 4.9. Statistical Analysis

Cold treatment was repeated at least three times, with three replicates for each line and time point. All data, expressed as mean ± SD, were analyzed by SPSS 25.0 software (SPSS Inc., Chicago, IL, USA). Analysis of variance (ANOVA) was used to compare the statistical difference based on Fisher’s least significant difference test at the significance levels of *p* < 0.05 (*), *p* < 0.01 (**), and *p* < 0.001 (***).

## 5. Conclusions

In the present study, cold treatment induced lignin accumulation, an increase in the laccase activity, and the differential regulation of 21 *CsLACs* in *C. sinensis* leaves. *CsLAC18* was one of the most significantly upregulated genes. CsLAC18 was localized in the cell membrane, and was phylogenetically related to lignin-specific AtLAC17. Functional analyses by overexpression and VIGS indicate that *CsLAC18* contributes to cold tolerance. As a whole, our results suggested that *CsLAC18* could be a potential target for improving resistance to cold stress in citrus.

## Data Availability

Not applicable.

## Supplementary Materials

The following supporting information can be downloaded at: https://www.mdpi.com/article/10.3390/ijms232314509/s1.

## References

[1] Food and Agriculture Organization of the United Nations. https://www.fao.org/faostat/en/.

[2] Primo-Capella A., Martínez-Cuenca M.-R., Forner-Giner M.Á. (2021). Cold stress in Citrus: A molecular, physiological and biochemical perspective. Horticulturae.

[3] Zhang S.Q., Liang M., Wang N., Xu Q., Deng X.X., Chai L.J. (2018). Reproduction in woody perennial Citrus: An update on nucellar embryony and self-incompatibility. Plant Reprod..

[4] Janusz G., Pawlik A., Swiderska-Burek U., Polak J., Sulej J., Jarosz-Wilkolazka A., Paszczynski A. (2020). Laccase properties, physiological functions, and evolution. Int. J. Mol. Sci..

[5] Yoshida H. (1883). LXIII.-Chemistry of lacquer (Urushi). Part I. Communication from the Chemical Society of Tokio. J. Am. Chem. Soc..

[6] Zaman F., Zhang M., Liu Y., Wang Z.L., Xu L.Q., Guo D.Y., Luo Z.R., Zhang Q.L. (2022). DkmiR397 regulates proanthocyanidin biosynthesis via negative modulating *DkLAC2* in Chinese PCNA persimmon. Int. J. Mol. Sci..

[7] Yu Y., Li Q.F., Zhang J.P., Zhang F., Zhou Y.F., Feng Y.Z., Chen Y.Q., Zhang Y.C. (2017). *Laccase-13* regulates seed setting rate by affecting hydrogen peroxide dynamics and mitochondrial integrity in rice. Front. Plant Sci..

[8] Wang J.H., Feng J.J., Jia W.T., Chang S., Li S.Z., Li Y.X. (2015). Lignin engineering through laccase modification: A promising field for energy plant improvement. Biotechnol. Biofuels.

[9] Zhao Y.C., Liu Y.Q., Dong X., Liu J.J., Zhao D.G. (2022). Identification of a novel laccase gene *EuLAC1* and its potential resistance against *Botrytis cinerea*. Transgenic Res..

[10] Liu J.J., Zhuang Y., Huang X.Z., Zhao D.G., Zhao Y.C. (2020). Overexpression of cotton laccase gene *LAC1* enhances resistance to *Botrytis cinereal* in tomato plants. Int. J. Agric. Biol..

[11] Zhang Y., Wu L.Z., Wang X.F., Chen B., Zhao J., Cui J., Li Z.K., Yang J., Wu L.Q., Wu J.H. (2019). The cotton laccase gene *GhLAC15* enhances Verticillium wilt resistance via an increase in defence-induced lignification and lignin components in the cell walls of plants. Mol. Plant Pathol..

[12] Hu Q., Min L., Yang X.Y., Jin S.X., Zhang L., Li Y.Y., Ma Y.Z., Qi X.W., Li D.Q., Liu H.B. (2018). Laccase GhLac1 modulates broad-spectrum biotic stress tolerance via manipulating phenylpropanoid pathway and jasmonic acid synthesis. Plant Physiol..

[13] Huang J.H., Zhang L.Y., Lin X.J., Gao Y., Zhang J., Huang W.L., Zhao D., Ferrarezi R.S., Fan G.C., Chen L.S. (2022). *CsiLAC4* modulates boron flow in *Arabidopsis* and *Citrus* via high-boron-dependent lignification of cell walls. New Phytol..

[14] Liu Q.Q., Luo L., Wang X.X., Shen Z.G., Zheng L.Q. (2017). Comprehensive analysis of rice laccase gene (OsLAC) family and ectopic expression of *OsLAC10* enhances tolerance to copper stress in *Arabidopsis*. Int. J. Mol. Sci..

[15] Cho H.Y., Lee C., Hwang S.G., Park Y.C., Lim H.L., Jang C.S. (2014). Overexpression of the *OsChI1* gene, encoding a putative laccase precursor, increases tolerance to drought and salinity stress in transgenic *Arabidopsis*. Gene.

[16] Ma J., Xu Z.S., Wang F., Xiong A.S. (2015). Isolation, purification and characterization of two laccases from carrot (Daucus carota L.) and their response to abiotic and metal ions stresses. Prot. J..

[17] Wan F.X., Zhang L.Q., Tan M.Y., Wang X.H., Wang G.L., Qi M.R., Liu B.X., Gao J., Pan Y., Wang Y.Q. (2022). Genome-wide identification and characterization of laccase family members in eggplant (*Solanum melongena* L.). Peerj.

[18] Xu X.Y., Zhou Y.P., Wang B., Ding L., Wang Y., Luo L., Zhang Y.L., Kong W.W. (2019). Genome-wide identification and characterization of laccase gene family in *Citrus Sinensis*. Gene.

[19] Lu C.Y., Yang T.Y., Zhang Y.W., Miao X.C., Jin C., Xu X.Y. (2021). Genome-wide analyses and expression patterns under abiotic stress of LAC gene family in pear (*Pyrus bretschneideri*). Plant Biotechnol. Rep..

[20] Cesarino I. (2019). Structural features and regulation of lignin deposited upon biotic and abiotic stresses. Curr. Opin. Biotechnol..

[21] Anderson M.D., Prasad T.K., Stewart C.R. (1995). Changes in isozyme profiles of catalase, peroxidase, and glutathione reductase during acclimation to chilling in mesocotyls of maize seedlings. Plant Physiol..

[22] Zhou P.L., Li Q.Y., Liu G.L., Xu N., Yang Y.J., Zeng W.L., Chen A.G., Wang S.S. (2019). Integrated analysis of transcriptomic and metabolomic data reveals critical metabolic pathways involved in polyphenol biosynthesis in *Nicotiana tabacum* under chilling stress. Funct. Plant Biol..

[23] Hu J.J., Wu W., Cao Z.H., Wen J., Shu Q.L., Fu S.L. (2016). Morphological, physiological and biochemical responses of *Camellia oleifera* to low-temperature stress. Pak. J. Bot..

[24] Khaledian Y., Maali-Amiri R., Talei A. (2015). Phenylpropanoid and antioxidant changes in chickpea plants during cold stress. Russ. J. Plant Physiol..

[25] dos Santos A.B., Bottcher A., Vicentini R., Sampaio Mayer J.L., Kiyota E., Landell M.A.G., Creste S., Mazzafera P. (2015). Lignin biosynthesis in sugarcane is affected by low temperature. Environ. Exp. Bot..

[26] Zagoskina N.V., Olenichenko N.A., Klimov S.V., Astakhova N.V., Zhivukhina E.A., Trunova T.I. (2005). The effects of cold acclimation of winter wheat plants on changes in CO_2_ exchange and phenolic compound formation. Russ. J. Plant Physiol..

[27] Hausman J.F., Evers D., Thiellement H., Jouve L. (2000). Compared responses of poplar cuttings and in vitro raised shoots to short-term chilling treatments. Plant Cell Rep..

[28] Cabane M., Afif D., Hawkins S. (2012). Lignins and Abiotic Stresses. Lignins-Biosynthesis, Biodegradation and Bioengineering: Advances in Botanical Research.

[29] Ping X.K., Wang T.Y., Lin N., Di F.F., Li Y.Y., Jian H.J., Wang H., Lu K., Li J.N., Xu X.F. (2019). Genome-wide identification of the LAC gene family and its expression analysis under stress in Brassica napus. Molecules.

[30] Li R., Zhao Y., Sun Z., Wu Z.Y., Wang H.L., Fu C.X., Zhao H.B., He F. (2022). Genome-wide identification of switchgrass laccases involved in lignin biosynthesis and heavy-metal responses. Int. J. Mol. Sci..

[31] Yu Y.C., Xing Y.X., Liu F.J., Zhang X., Li X.W., Zhang J., Sun X.L. (2021). The laccase gene family mediate multi-perspective trade-offs during tea plant (*Camellia sinensis*) development and defense processes. Int. J. Mol. Sci..

[32] Sun Z.X., Zhou Y.L., Hu Y., Jiang N., Hu S.J., Li L., Li T. (2022). Identification of wheat *LACCASEs* in response to *Fusarium graminearum* as potential deoxynivalenol trappers. Front. Plant Sci..

[33] Qui K.L., Zhou H., Pan H.F., Sheng Y., Yu H., Xie Q.M., Chen H.L., Cai Y.P., Zhang J.Y., He J.L. (2022). Genome-wide identification and functional analysis of the peach (*P. persica*) laccase gene family reveal members potentially involved in endocarp lignification. Trees.

[34] Berthet S., Demont-Caulet N., Pollet B., Bidzinski P., Cezard L., Le Bris P., Borrega N., Herve J., Blondet E., Balzergue S. (2011). Disruption of *LACCASE4* and *17* results in tissue-specific alterations to lignification of *Arabidopsis thaliana* stems. Plant Cell.

[35] Wang Y., Bouchabke-Coussa O., Lebris P., Antelme S., Soulhat C., Gineau E., Dalmais M., Bendahmane A., Morin H., Mouille G. (2015). LACCASE5 is required for lignification of the *Brachypodium distachyon* Culm. Plant Physiol..

[36] Cesarino I., Araujo P., Sampaio Mayer J.L., Vicentini R., Berthet S., Demedts B., Vanholme B., Boerjan W., Mazzafera P. (2013). Expression of *SofLAC*, a new laccase in sugarcane, restores lignin content but not S:G ratio of *Arabidopsis lac17* mutant. J. Exp. Bot..

[37] Fang F., Zhang X.L., Luo H.H., Zhou J.J., Gong Y.H., Li W.J., Shi Z.W., He Q., Wu Q., Li L. (2015). An intracellular laccase Is responsible for epicatechin-mediated anthocyanin degradation in litchi fruit pericarp. Plant Physiol..

[38] Wang P., Zhang B., Li X., Xu C.J., Yin X.R., Shan L.L., Ferguson I., Chen K.S. (2010). Ethylene signal transduction elements involved in chilling injury in non-climacteric loquat fruit. J. Exp. Bot..

[39] Wang M., Dai W.S., Du J., Ming R.H., Dahro B., Liu J.H. (2019). ERF109 of trifoliate orange (*Poncirus trifoliata* (L.) Raf.) contributes to cold tolerance by directly regulating expression of *Prx1* involved in antioxidative process. Plant Biotechnol. J..

[40] Fu X.Z., Chen C.W., Wang Y., Liu J.H., Moriguchi T. (2011). Ectopic expression of *MdSPDS1* in sweet orange (*Citrus sinensis* Osbeck) reduces canker susceptibility: Involvement of H_2_O_2_ production and transcriptional alteration. BMC Plant Biol..

[41] Zhang Y.L., Zhang Y.W., Luo L., Lu C.Y., Kong W.W., Cheng L.B., Xu X.Y., Liu J.H. (2022). Genome wide identification of *respiratory burst oxidase homolog* (*Rboh*) genes in *Citrus sinensis* and functional analysis of *CsRbohD* in cold tolerance. Int. J. Mol. Sci..

[42] Shi J., Fu X.Z., Peng T., Huang X.S., Fan Q.J., Liu J.H. (2010). Spermine pretreatment confers dehydration tolerance of citrus in vitro plants via modulation of antioxidative capacity and stomatal response. Tree Physiol..

[43] Huang X.S., Wang W., Zhang Q., Liu J.H. (2013). A basic helix-loop-helix transcription factor, *PtrbHLH*, of *Poncirus trifoliata* confers cold tolerance and modulates peroxidase-mediated scavenging of hydrogen peroxide. Plant Physiol..

