# Supplementary material for: The Citrus Laccase Gene CsLAC18 Contributes to Cold Tolerance
Source: Int J Mol Sci. 2022 Nov 22;23(23):14509. doi: 10.3390/ijms232314509 (PMC9737282; doi:10.3390/ijms232314509)
Supplement: Supplementary file 1 [file ijms-23-14509-s001.zip › Supplementary Figure S1-S5.pdf]

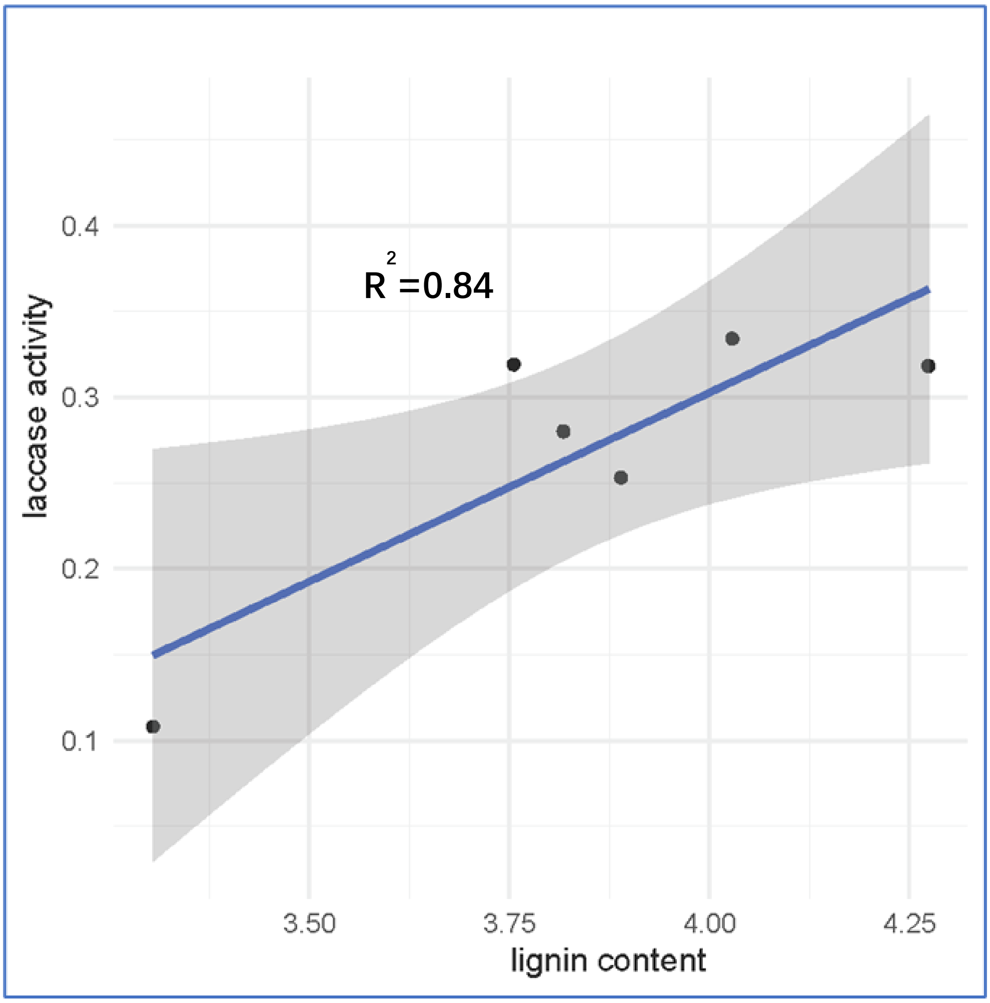

Supplementary Figure S1. Correlation between laccase activity and lignin content.

> CsLAC18
ATGGGAGCTTCTCTTCTTCGATCACCAGCATTTCTAGGAGTCATTTGCTCATTTATCAAC
TTGTGTCTGCTTGCTGAGCCTGCACTCGGTGTCACCAGGCACTACAAGTTTGATATCAAG
TTGCAAAATGTGACGCGTCTTTGTAACACCAAGAGCATTATATCAGTAAATGGGAAGTTT
CCAGGGCCTCGCATTGTAGCAAGGGAGGGTGACCAGCTTCTTATCAAAGTTATGAACCAT
GTCCAGAACAATATTTCCATCCATTGGCATGGAATTAGACAGCTTCGAAGTGGATGGGCC
GACGGACCAGCTTATATTACTCAATGCCCCATTCAAACAGGGCAGAGCTACGTTTACAAC
TTCACCATTGTTGGCCAAAGAGGAACACTCTGGTGGCATGCTCACTTATCGTGGCTACGA
TCAACTCTCTATGGTCCCATCATCATTCTTCCCAAGCGTGGCATTCCTTACCCATTTACC
AAGCCTTACAAGGAAGTTCCCATTATCTTTGGAGAGTGGTTCAAAGCAGATCCTGAGACT
ATCATTAGCCAGGCCCTACAAACAGGTGGAGGCCCAAATGTATCTGATGCATATACCATC
AATGGACTCCCAGGGCCATTGTATAACTGCTCTGCCAAAGACACATTCAAGCTGAAGGTG
AAGCCCGGAAAAACTTACCTTCTCCGGTTAATCAATGCTGCACTGAATGACGAGCTCTTC
TTCAGCATAGCAAACCACACCCTTACAGTTGTTGAAGCTGATGCTATTTACGTTAAACCT
TTTGAAACTGAAACACTACTCATTGCCCCTGGACAGACAACGAATGTTCTTCTCAAAACA
AAACCTCACTACCCAAGTGCCACATTTTTCATGAAAGCTAGACCTTATGTAACTGGCCAG
GGCACTTTCGACAATTCAACCGTTGCTGGTATCTTAGAGTATGAAAAACCACTCAATTTC
CATCTTTCAAGCAACTCCATTAAAAATCTTCCCCTCTTCAAACCAGTTCTACCTGCTCTC
AATGACACTTCCTTTGCTACAAGTTTTACAAATAAGCTTCGTAGCTTAGCAAGCACACAG
TTTCCTGCCAATGTGCCCCAGAATGTTGATAGGCGATTTTTCTTCACGGTAGGCCTAGGA
ACAAGCCCCTGCCAGAGTAACCAAACCTGCCAAGGTCCCAATGGAACCATGTTTCAAGCT
TCAGTCAATAACATTTCTTTTGTAATGCCAACCACAGCTCTACTCCAAGCTCACTTTACT
GGAAAATCAGATGGTGTTTACACCCCTGATTTTCCTACCAGTCCATTGATTGCATTTAAT
TATACGGGCACTCCACCCAATAACACGATGGTAAGCAATGGAACAAAGCTAGTGGTGCTT
CCTTTTAACTCTAGTGTGGAGCTAATAATGCAGGATACAAGCATTCTTGGAGCTGAGAGC
CACCCTCTTCACTTGCATGGCTTCAATTTCTTCGTTGTCGGCCAAGGTTTTGGAAATTTC
GATCCAAATAAGGACCCTACAAAATTTAACCTTGTCGATCCCGTTGAAAGGAACACAGTT
GGCGTGCCCTCGGGTGGCTGGGTAGCAATTCGGTTCCTTGCAGATAATCCAGGAGTATGG
TTTATGCATTGCCACCTTGAAGTGCATACAAGCTGGGGTCTGAAGATGGCTTGGATTGTT
TTAGATGGAAAGCATCCTAATCAGAAGCTACCCCCTCCACCAGCAGATCTTCCTCAGTGC
TGA

Supplementary Figure S2. The full-length cDNA sequences of *CsLAC18* from *C. sinensis*.

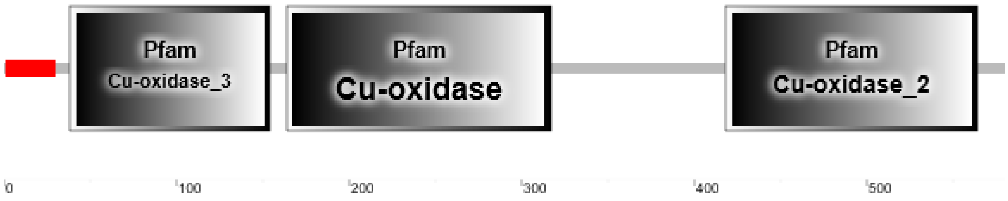

Supplementary Figure S3. Domain compositions of *CsLAC18* proteins by SMART analysis. Red box in the figure indicates a signal peptide.

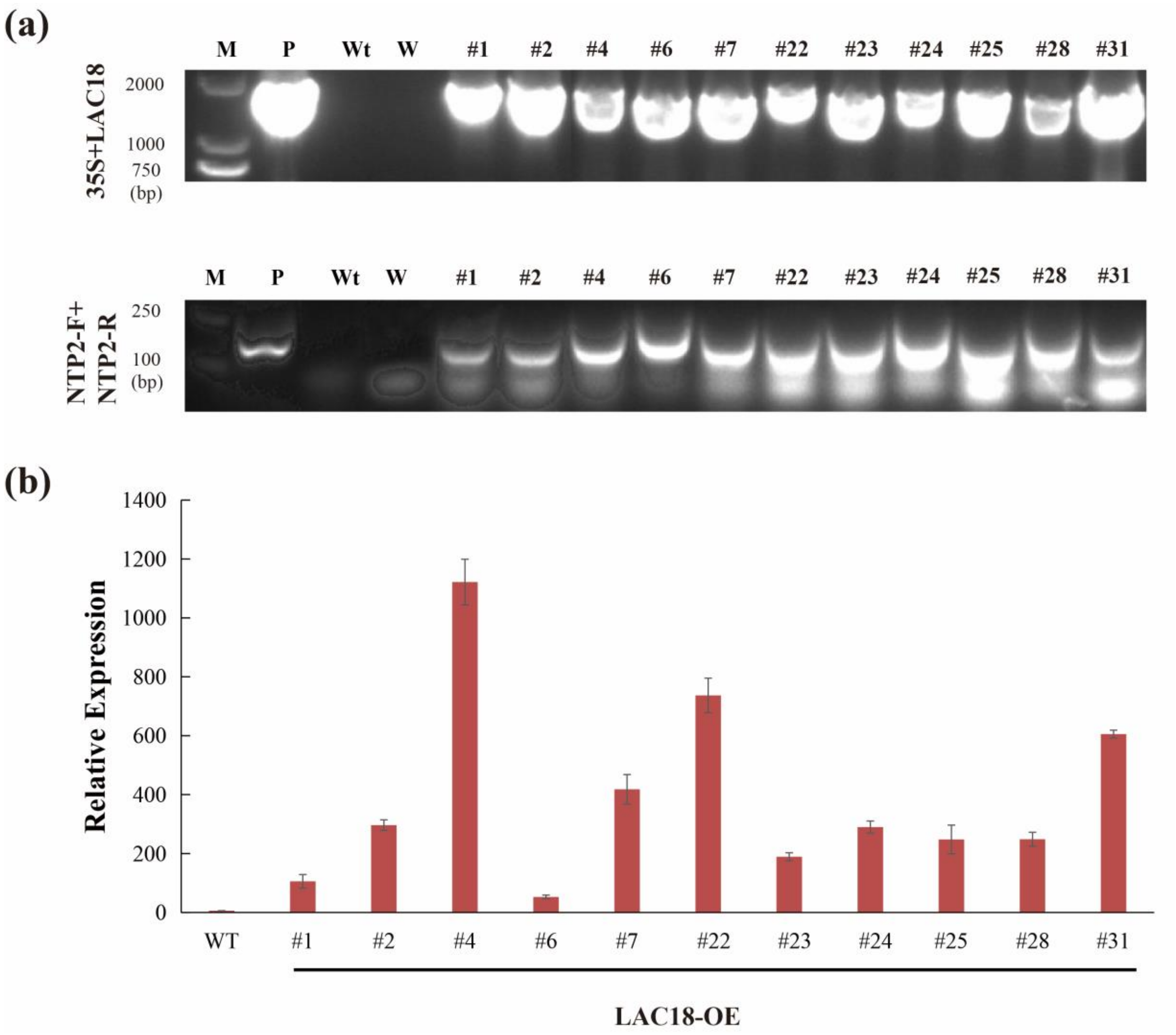

Supplementary Figure S4. Molecular identification of transgenic tobacco plants overexpressing *CsLAC18*. (a) Characterization of transgenic tobacco plants by genomic PCR amplification. (b) Analysis of *CsLAC18* expression in tobacco wild type (WT) and transgenic plants using qPCR.

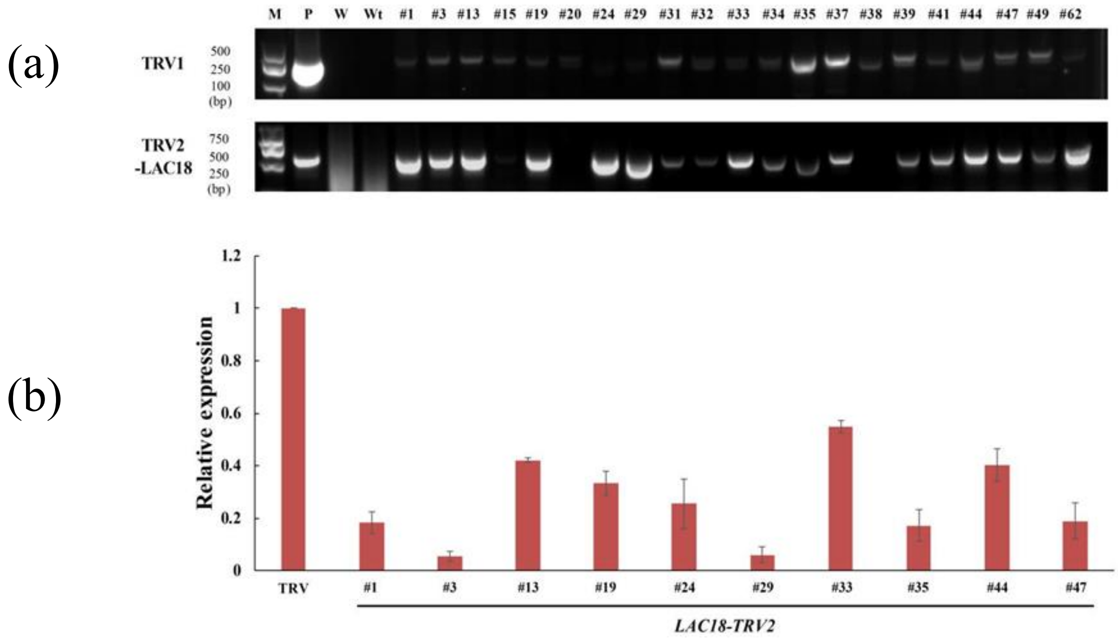

Supplementary Figure S5. Molecular characterization of the VIGS plants by genomic PCR and qRT-PCR. (a) Characterization of positive VIGS plants by genomic PCR amplification. (b) Analysis of *CsLAC18* expression in the control (TRV) and VIGS plants using qRT-PCR.
